# Distilling data - A flexible method for producing research-ready Electronic Health Records

**DOI:** 10.23889/ijpds.v1i1.367

**Published:** 2017-04-19

**Authors:** Alex Hacker

**Affiliations:** 1 The Nuffield Department of Population Health

## Objectives

Population health datasets need to combine data from multiple sources into a coherent whole. This presents significant technical challenges: both in standardising the data, and in presenting it in a form suitable for research. This presentation outlines a method that we have developed for managing the Electronic Health Records (EHRs) gathered by our biobank, and for aggregating them without obscuring important features. The technique is broadly applicable, illustrated with practical examples derived from our dataset of >1.7 million health events.

## Approach

We begin with a disparate set of EHRs from different sources: death certificates, a national disease registry, and multiple Health Insurance agencies.

Endpoint definitions - We first specify our outcomes of interest. Generally, these simply consist of a list of the ICD10 codes that define a disease, which could be as broad as 'vascular disease' or as narrow as 'macrovascular complications of diabetes'.

Events list - Then we standardise the required EHR variables. Typically these are just the diagnoses and the dates. Our standardisation methods necessarily vary by data source, but the goal is the same for each: a list of dated diagnoses in ICD10 format.

Endpoints - Finally, we automatically compare our definitions and events to determine whether each participant has experienced any of the endpoints and, if so, when their earliest occurrence was.

## Results

The resulting endpoint dataset has many desirable properties. For example, analyses are simplified because each participant has a single value for each endpoint, regardless of the number of events that they have. Immediately we can answer common questions, such as 'How many of our participants have had a stroke, and how soon after we gathered their physical measurements?'

We retain the source of each event, so we can compare sources to check internal validity, or restrict our endpoints to only consider fatal events, for example. New events, and even entirely new sources of events, can be incorporated once the key variables are standardised. New endpoints can be created directly from their ICD10 definition.

More complex endpoints can be produced from any suitable variable. Second stroke, revascularisation surgery, antidepressant use: once we have the definition & data, a suitable endpoint can be created in exactly the same easy-to-use format.

## Conclusion

This methodology facilitates validation, comparison and combination of data sources. It enables us to present complex EHR data in a clear and flexible form, allowing researchers to analyse it with ease and confidence.

